# Melanoma Treatments and Mortality Rate Trends in the US, 1975 to 2019

**DOI:** 10.1001/jamanetworkopen.2022.45269

**Published:** 2022-12-06

**Authors:** Navkirat Kahlon, Sishir Doddi, Rame Yousif, Sana Najib, Taha Sheikh, Ziad Abuhelwa, Cameron Burmeister, Danae M. Hamouda

**Affiliations:** 1 Division of Hematology and Oncology, Department of Medicine, University of Toledo College of Medicine and Life Sciences, Toledo, Ohio; 2 Department of Medicine, University of Toledo College of Medicine and Life Sciences, Toledo, Ohio

## Abstract

**Importance:**

Melanoma accounts for most of the deaths due to skin cancer. In the past decade, effective US Food and Drug Administration (FDA)–approved therapies for melanoma have emerged.

**Objective:**

To review changes in the long-term melanoma mortality rate (MMR) trends in the US and determine whether they have any temporal association with the FDA approval of new agents.

**Design, Setting, and Participants:**

This cross-sectional study used population data from the Surveillance, Epidemiology, and End Results (SEER) database and retrospectively reviewed the age-adjusted MMR trends in adult patients (aged ≥18 years) from 1975 to 2019 in the US population. The timeline of the FDA approvals for melanoma treatment was also reviewed. Data were analyzed from March 15 to August 15, 2022.

**Exposures:**

Outcomes were assessed in association with FDA approval of drugs for the treatment of melanoma.

**Main Outcomes and Measures:**

Mortality rates are from the SEER database, reported per 100 000 population and age-adjusted to the 2000 US standard population. The annual percent change (APC) has been used to report long-term trends.

**Results:**

After the introduction of newer treatments in 2011 (most after 2013), a significant reduction in MMR was seen from 2013 to 2017 in the US for the first time in the past 40 years. Rates increased from 1975 to 1988 (APC, 1.65% [95% CI, 1.30%-2.00%]; *P* < .001). No statistically significant change in MMR was seen from 1988 to 2013 (APC, 0.01% [95% CI, −1.10% to 0.12%]; *P* = .85). The MMR decreased significantly from 2013 to 2017 (APC, −6.28% [95% CI, −8.52% to −3.97%]; *P* < .001).

**Conclusions and Relevance:**

These findings suggest a benefit associated with the availability of effective therapies in the past decade and further suggest that the use of new pharmacological therapies is associated with decreased MMR in the US population. These data are very encouraging and support the continued development of such therapies. Additionally, the accessibility of these treatments and the associated health care costs need to be addressed.

## Key Points

**Question:**

Are new treatments for cutaneous melanoma associated with changes in melanoma mortality rate (MMR) trends among US adults?

**Findings:**

In this population-based cross-sectional study of patients with cutaneous melanoma from the Surveillance, Epidemiology, and End Results database from 1975 to 2019, the MMR decreased from 2013 to 2017, since the availability of new and effective treatment options after 2011.

**Meaning:**

These findings suggest the trend of decreased MMR is temporally associated with US Food and Drug Administration approval of new and effective treatment options for melanoma.

## Teaser

This cross-sectional study uses the Surveillance, Epidemiology, and End Results database to identify trends in the melanoma mortality rate among US adults from 1975 and 2019 and assess whether a change in the epidemiology of melanoma is associated with the advent of new and effective treatment options.

## Introduction

Melanoma is the deadliest skin cancer, historically known for its extremely poor prognosis after metastasis.^1,2^ The incidence of melanoma has continued to rise.^3,4^ Increasing incidence has been attributed to skin screening programs, but they do not decrease mortality.^5,6,7^ Melanoma globally causes an estimated 57 000 deaths annually.^8,9^ The traditional approach for treating metastatic melanoma was sequential chemotherapy, which was associated with little overall survival benefit.^10^ In the past decade, new and effective therapies for melanoma have emerged, with US Food and Drug Administration (FDA) approval of multiple checkpoint inhibitors such as programmed cell death protein 1 (PD-1) and cytotoxic T-lymphocyte–associated antigen 4 (CTLA-4) inhibitors and multiple tyrosine kinase inhibitors targeting v-RAF murine sarcoma viral oncogene homologue B1 (*BRAF*)–mutated melanoma.^11,12^ These therapies have demonstrated remarkably improved overall survival in pivotal clinical trials.

Historically, the prognosis of metastatic melanoma with cytotoxic chemotherapy has been poor. However, with the use of novel therapies, the 5-year overall survival rate has increased from 5% to greater than 50%.^13,14^ A frequent cause of mortality in patients with metastatic melanoma is brain metastases, with more than 50% of patients developing these metastases in their lifetime.^15,16^ The median overall survival from the diagnosis of brain metastases historically has been approximately 5 months; however, current therapies have extended the median overall survival in these patients to approximately 3 years.^15^ The use of dual immunotherapy, such as ipilimumab-nivolumab, has shown an overall 5-year survival rate greater than 50%, albeit at the cost of high levels of treatment toxicity.^17^ Recently, the FDA approved nivolumab and relatlimab, a lymphocyte activation gene 3 (LAG-3)–blocking antibody, for patients with advanced melanoma, which seems to have a better toxicity profile.^18^

Given the magnitude of benefit from these treatments compared with traditional chemotherapy, one can anticipate a potential association with improved mortality outcomes.^11^ This study used the Surveillance, Epidemiology, and End Results (SEER) database to identify trends in the melanoma mortality rate (MMR) in the US population during the past 4 decades (1975-2019) to determine whether a change in the epidemiology of melanoma (specifically MMR) is associated with the advent of these new and effective treatment options.

## Methods

This cross-sectional study followed the Strengthening the Reporting of Observational Studies in Epidemiology (STROBE) reporting guideline. Our analysis is derived from retrospective SEER data, which is publicly available and anonymized. The links to access these data are provided in the references. Therefore, institutional review board approval and patient consent were not required according to the Common Rule, as we used publicly available deidentified data under a data use agreement with the US National Cancer Institute.

### Data Source 

Long-term age-adjusted cutaneous MMR trends in the US using the SEER database from 1975 to 2019 were reviewed to determine any temporal association with the FDA approval of effective treatments.^19^ The inclusion criteria for patients consisted of being 18 years or older and having a diagnosis of cutaneous melanoma. Data for sex (men and women) and race and ethnicity (Black, White, and other race or ethnicity) were collected from the SEER database. Race and ethnicity data were collected to identify any racial or ethnic disparity trends for mortality outcomes. The National Cancer Institute’s SEER Program publishes cancer statistics in the US. Cancer sites are defined using the SEER Cause of Death Recode 1969+ (04/16/2012).^20^

### Statistical Analysis

Data were analyzed from March 15 to August 15, 2022. Mortality rates reported in the SEER database are per 100 000 population and age-adjusted to the 2000 US standard population. The source of mortality data for SEER is the US Mortality Files, National Center for Health Statistics, Centers for Disease Control and Prevention. The annual percent change (APC) with 95% CI and *P* value has been used to report long-term trends. The APC estimates reported in the SEER Explorer Program have been calculated from the underlying rates using the Joinpoint Trend Analysis Software, version 4.9.1.0.^21^ Two-sided *P* ≤ .05 was considered to indicate a statistically significant difference. The timeline for FDA approvals for melanoma treatment was reviewed to determine any temporal association with MMR trends.

## Results

The MMR showed a downtrend during the period between 2013 and 2017 for the first time in the past 40 years. The MMR rate per 100 000 US population was 2.07 (95% CI, 2.00-2.13) in 1975, 2.65 (95% CI, 2.58-2.65) in 1988, 2.67 (95% CI, 2.61-2.72) in 2013, 2.09 (95% CI, 2.05-2.14) in 2017, and 2.01 (95% CI, 1.97-2.06) in 2019. The MMR increased from 1975 to 1988 (APC, 1.65% [95% CI, 1.30%-2.00%]; *P* < .001). In the 1970s, chemotherapy agents were introduced for melanoma but, with their marginal efficacy, no mortality benefit in the US population was seen. No statistically significant change in MMR was seen between 1988 and 2013 (APC, 0.01% [95% CI, −1.10% to 0.12%]; *P* = .85). During this period, no breakthrough treatment options emerged until the introduction of checkpoint inhibitors (ipilimumab) and *BRAF* inhibitors (vemurafenib) in 2011. Gradually, immunotherapy treatments became part of the treatment paradigm, given unprecedented responses in heavily pretreated patients who historically had a dismal prognosis. With the introduction of many more agents and combination treatments since 2013, the MMR significantly decreased from 2013 to 2017 (APC, −6.28% [95% CI, −8.52% to −3.97%]; *P* < .001). This statistically significant reduction in mortality during this period was seen across all the subgroups regardless of race, age group, or sex.^19^ No statistically significant decrease was seen from 2017 to 2019 (APC, −1.56% [95% CI, −6.41% to 3.55%]; *P* = .53); however, the trend was downward. The MMR trends are summarized in Table 1. We reviewed the timeline of FDA approvals for melanoma treatment, and the advent of new therapies appeared to have a temporal association with decreasing MMR trends. These results are summarized in Table 2 and Table 3.

**Table 1.** Long-term Trends in US Age-Adjusted Melanoma Mortality Rates, 1975-2019^19^

Year range	APC, % (95% CI)	*P* value	Interpretation
1975-1988	1.65 (1.30 to 2.00)	<.001	Increasing
1988-2013	0.01 (−1.10 to 0.12)	.85	Stable
2013-2017	−6.28 (−8.52 to −3.97)	<.001	Decreasing
2017-2019	−1.56 (−6.41 to 3.55)	.53	No statistically significant change

Abbreviation: APC, annual percent change.

**Table 2.** FDA-Approved Therapies for Metastatic or Unresectable Stage III Melanoma Since 2011 and a Brief Summary of Pivotal Trials Leading to Corresponding FDA Approval, Focusing on OS Outcomes

Therapy	FDA approval year	Dose	Comparator	Trial name	Study phase	Primary end point	Statistically significant OS benefit	Indication or line of treatment in patients with metastatic or unresectable melanoma	Statistically and clinically significant outcomes from trials
Ipilimumab^12^	2011	3 mg/kg body weight IV every 3 wk	Gp100	NA	3	OS	Yes	First line	Median OS 10.1 mo with ipilimumab vs 6.4 mo with Gp100
Vemurafenib^22^	2011	960 mg PO BID	Dacarbazine	BRIM-3	3	OS and PFS	Yes	Melanoma with *BRAF* V600E mutation (first line)	Median OS 13.6 mo with vemurafenib vs 9.7 mo with dacarbazine
Dabrafenib mesylate^23,24,25^	2013	150 mg PO BID	Dacarbazine	BREAK-3	3	ORR and OS	Yes	Metastatic melanoma with *BRAF* V600E mutation (first line)	5-y OS 24% with dabrafenib vs 22% with dacarbazine, but crossover to dabrafenib was allowed
Trametinib dimethyl sulfoxide^26^	2013	2 mg PO once a day	Dacarbazine or paclitaxel	METRIC	3	PFS and OS	Yes	Melanoma with *BRAF* V600 mutation (first line)	6-mo OS 81% with trametinib vs 67% with dacarbazine or paclitaxel, including 47% patients with crossover to trametinib after progression
Pembrolizumab^27,28^	2014	10 mg/kg body weight every 2 or 3 wk	Ipilimumab (3 mg/kg) every 3 wk for 4 doses	KEYNOTE 006	3	PFS and OS	Yes	First line	Median OS 32.7 mo with pembrolizumab vs 15.9 mo with ipilimumab
Nivolumab monotherapy^29,30^	2014	3 mg/kg body weight IV every 2 wk	Investigator's choice chemothrapy (dacarbazine, 1000 mg/m^2^ every 3 wk, or paclitaxel, 175 mg/m^2^, combined with carboplatin [area under the curve, 6] every 3 wk)	CheckMate 037	3	Objective response	No	Second line (refractory to ipilimumab or a *BRAF* inhibitor)	Approval based on better response rate 32% with nivolumab vs 11% with chemotherapy
Nivolumab monotherapy^31,32^	2015	3 mg/kg body weight IV every 2 wk	Dacarbazine	CheckMate 066	3	PFS and OS	Yes	Metastatic melanoma (first line)	5-y OS 39% with nivolumab vs 17% with dacarbazine
Nivolumab with ipilimumab^33,34^	2015	Nivolumab, 1 mg/kg every 2 wk, plus ipilimumab, 3 mg/kg every 2 wk, for 4 doses, followed by nivolumab, 3 mg/kg every 2 wk	Nivolumab (3 mg/kg every 2 or 3 wk) or ipilimumab (3 mg/kg every 3 wk for 4 doses)	CheckMate 067	3	PFS and OS	Yes	Metastatic melanoma (first line)	Median OS 72.1 mo in combination vs 36.9 mo with nivolumab vs 19.9 mo with ipilimumab
Dabrafenib with trametinib^35,36,37^	2015	Dabrafenib, 150 mg PO BID with trametinib, 2 mg once a day	Dabrafenib	COMBI-d	3	PFS	Yes	Melanoma with *BRAF* V600 mutation (first line)	Median OS 25.1 mo with combination vs 18.7 mo with dabrafenib; 3-y OS 45% with combination vs 32% with vemurafenib
			Vemurafenib	COMBI-v					
Vemurafenib plus cobimetinib fumarate^38,39^	2015	Vemurafenib, 960 mg PO BID, and cobimetinib fumarate, 60 mg once a day	Vemurafenib	coBRIM	3	PFS	Yes	Melanoma with *BRAF* V600 mutation (first line)	5-y OS 31% with combination vs 26% with vemurafenib
Talimogene laherparepvec^40,41,42^	2015	Intralesional first dose, 10^6^ pfu/mL, then 10^8^ pfu/mL every 2 wk starting 3 wk after first dose	GM-CSF	OPTiM	3	OS and DRR	No	Unresectable stage III or IV melanoma (first line)	DRR (>6 mo) 16.3% with talimogene laherparepvec vs 2.1% with GM-CSF
Encorafenib plus binimetinib^43^	2018	Encorafenib, 450 mg PO once a day, plus binimetinib, 45 mg PO BID	Vemurafenib	COLUMBUS	3	PFS	Yes	Metastatic or unresectable melanoma with *BRAF* V600 mutation (first line)	Median OS 33.6 mo with combination vs 16.7 mo with vemurafenib
Atezolizumab plus cobimetinib and vemurafenib^44^	2020	Cobimetinib, 60 mg PO once a day (d 1-21), plus vemurafenib, 720 mg PO BID plus IV atezolizumab, 840 mg, d 1 plus 15 d in 28-d cycles	Placebo with cobietinib and vemurafenib	IMspire150	3	OS and PFS	No	Metastatic melanoma with *BRAF* V600 mutation (first line)	OS data not mature yet, approval based on PFS of 15.1 mo with combination vs 10.6 mo with placebo and cobietinib and vemurafenib
Relatlimab and nivolumab^18^	2022	Relatlimab, 160 mg IV, and nivolumab, 480 mg IV in a fixed dose combination every 4 wk	Nivolumab, 480 mg IV every 4 wk	RELATIVITY-047	2/3	PFS	No	Unresectable stage III^a^ or metastatic melanoma (first line)	OS data not mature yet; median PFS 10.1 mo with combination vs 4.6 mo with nivolumab

Abbreviations: BID, twice daily; *BRAF*, v-RAF murine sarcoma viral oncogene homologue B1; DRR, durable response rate; FDA, US Food and Drug Administration; GM-CSF, granulocyte-macrophage colony-stimulating factor; Gp100, glycoprotein 100; IV, intravenously; NA, not applicable; ORR, overall response rate; OS, overall survival; PFS, progression-free survival; pfu, plaque-forming unit; PO, orally.

^a^ The stage of melanoma is per American Joint Committee on Cancer staging system for cancers.

**Table 3.** FDA-Approved Adjuvant Therapies for Melanoma Since 2011 and Summary of Pivotal Trials Leading to Corresponding FDA Approval

Therapy	FDA approval year	Dose (duration as mentioned below or until disease recurrence or medication toxicity)	Comparator	Trial	Phase	Primary end point	OS benefit (yes/no)	Indication/line of treatment	Statistically significant and clinically significant outcomes from trials
Pegylated interferon alfa-2b^45^	2011	6 μg/kg body weight SC weekly for 8 wk then 3 μg/kg SC weekly for 5 y	Placebo	EORTC 18991	3	RFS and OS	No	Stage III melanoma, microscopic or gross nodal involvement after surgery^a^	4-y RFS 45.6% with pegylated interferon alfa-2b vs 38.9% with placebo
Ipilimumab monotherapy^46,47,48^	2015	10 mg/kg body weight IV every 3 wk for 4 doses	Placebo	EORTC 18071	3	RFS and OS	Yes	Stage III melanoma, pathologic involvement of regional lymph nodes >1 mm after surgery	7-y OS rate of 60.0% with ipilimumab vs 51.3% with placebo; 1% deaths in ipilimumab group related to DRAE
Nivolumab monotherapy^49,50^	2017	3 mg/kg body weight IV every 3 wk for 4 doses	Ipilimumab	CheckMate 238	3	RFS and OS	No	Resected stage IIIC and IV melanoma	4-y RFS of 52% with nivolumab vs 41% with ipilimumab
Dabrafenib plus trametinib^51^	2017	Dabrafenib, 150 mg PO BID, plus trametinib, 2 mg PO BID for 12 mo	Placebo	COMBI-AD	3	OS	Yes	Stage III melanoma with *BRAF* V600E/V600K mutations	3-y OS rate of 86% with combination vs 77% with placebo
Pembrolizumab^52,53^	2019	200 mg IV every 3 wk for 18 cycles	Placebo	KEYNOTE-054	3	RFS	No	Completely resected stage III melanoma	3.5-y RFS 59.8% with pembrolizumab vs 41.1% with placebo
Pembrolizumab^54^	2021	200 mg IV every 3 wk for 17 cycles	Placebo	KEYNOTE-716	3	RFS and OS	No	Completely resected high-risk stage II melanoma	21-mo Follow-up for first recurrence of disease or mortality 15% with pembrolizumab vs 24% with placebo

Abbreviations: *BRAF*, v-RAF murine sarcoma viral oncogene homologue B1; DRAE, drug-related adverse events; EORTC, European Organisation for Research and Treatment of Cancer; FDA, US Food and Drug Administration; IV, intravenously; OS, overall survival; RFS, recurrence-free survival; SC, subcutaneously.

^a^ Stage of melanoma is per American Joint Committee on Cancer staging system for cancers.

Historically (before 2010), chemotherapy agents were sequenced to treat advanced cutaneous melanoma. The benefit was marginal, with rare complete responses and overall survival at 5 years ranging from 2% to 6%, and treatment was associated with significant toxic effects. Interleukin 2 was then approved in the 1990s with response rates less than 20%. In the early 2000s, different chemotherapeutic agents with some proven activity but without overall survival benefit were sequenced.^23^ No improvement in population mortality was seen during this period either.

Since 2011, several new agents have received FDA approval, more than ever before in melanoma. We will first discuss the timeline of drug approvals in metastatic disease and then the approvals in the adjuvant setting with a brief discussion about clinical data from the corresponding trials focusing primarily on overall survival.

Ipilimumab, an anti–CTLA-4 antibody, was approved by the FDA in 2011 for metastatic melanoma.^12^ The approval was based on results of a phase 3 trial involving patients with pretreated metastatic melanoma and showed an overall survival benefit compared with the glycoprotein 100 peptide vaccine (ie, 10.1 months with ipilimumab [3 mg/kg intravenously every 3 weeks] vs 6.4 months with glycoprotein 100; hazard ratio [HR], 0.66; *P* = .003).^12^

Vemurafenib, a *BRAF* kinase inhibitor, was approved by the FDA for metastatic melanoma with a *BRAF* V600E mutation in 2011 based on results of a phase 3 BRIM-3 trial comparing vemurafenib against dacarbazine.^22^ Overall survival was 84% (95% CI, 78%-89%) with vemurafenib and 64% (95% CI, 56%-73%) with dacarbazine. Even with the crossover to vemurafenib after progression with chemotherapy, the updated analysis continued to show overall survival benefit in the vemurafenib group (median, 13.6 vs 9.7 months; HR, 0.70; *P* < .001).^22^

Dabrafenib mesylate, a *BRAF* inhibitor, was approved by FDA in 2013 for the treatment of metastatic melanoma with a *BRAF* V600E mutation after results from the BREAK-3 study.^23,24^ Even after crossover to dabrafenib after progression with dacarbazine, dabrafenib has a slightly superior 5-year overall survival rate of 24% vs 22% after 5 years.^25^

Trametinib dimethyl sulfoxide, an allosteric inhibitor of MEK1 and MEK2 kinases, was approved in 2013 after the results of the METRIC trial for *BRAF* V600E– or *BRAF* V600K–positive advanced melanoma.^26^ Trametinib vs chemotherapy (paclitaxel or dacarbazine) showed a 6-month overall survival of 81% with trametinib vs 67% with chemotherapy (HR, 0.54; *P* = .01), even with 47% of patients receiving chemotherapy crossing over to trametinib treatment.^26^

In 2014, pembrolizumab, an anti–PD-1 antibody, was approved for treatment of advanced melanoma after showing superior progression-free survival (PFS) as well as overall survival and less high-grade toxicity in comparison with ipilimumab (3 mg/kg every 3 wk for 4 total doses) in the randomized phase 3 trial KEYNOTE 006.^27,28^ Updated results after 5 years of follow-up showed a median overall survival of 32.7 months in the pembrolizumab groups and 15.9 months in the ipilimumab group (HR, 0.73 [*P* < .001)].^27,28^

In 2014, nivolumab was approved as second-line therapy among patients with disease refractory to ipilimumab or a *BRAF* inhibitor for advanced melanoma after the positive results of the CheckMate 037 trial based on higher rates of response compared with the investigator’s choice of chemotherapy (ie, 32% vs 11%).^29,30^ The updated analysis published later does not show overall survival benefit.^29,30^

In 2015, the FDA approved nivolumab in untreated patients with metastatic melanoma without a *BRAF* mutation. Approval was based on the results of CheckMate 066 showing the overall survival and PFS benefit of nivolumab compared with dacarbazine.^31^ Updated 5-year overall survival rates continued to show superiority (ie, 39% vs 17%).^32^

In 2015, the results of the CheckMate 067 study led to the approval of the combination of nivolumab plus ipilimumab in patients with untreated advanced melanoma with and without *BRAF* V600 mutation.^33^ This trial compared the combination regimen vs nivolumab or ipilimumab monotherapy as the frontline treatment for these patients. The toxicity of the combination therapy, however, raised concerns. Drug-related adverse events (DRAE) of grade 3 or 4 occurred in 16.3% of patients in the nivolumab group and 55.0% of those in the nivolumab plus ipilimumab group. Updated results from a 6.5-year follow-up from this study showed a median overall survival of 72.1 months in the combination group, 36.9 months in the nivolumab group, and 19.9 months in the ipilimumab group.^34^

Also in 2015, results of 2 other phase 3 trials comparing dabrafenib plus trametinib (*BRAF* and MEK inhibitors) with dabrafenib monotherapy (COMBI-d) and vemurafenib monotherapy (COMBI-v) for the treatment of advanced melanoma with *BRAF* V600E or V600K mutations led to the approval of the combination therapy.^35,36^ In COMBI-d, the median overall survival was 25.1 months with the combination therapy and 18.7 months with dabrafenib monotherapy.^35^ In the COMBI-v study, the reported 3-year follow-up overall survival was 45% with combination therapy vs 32% with vemurafenib monotherapy.^36^ Pooled data from the COMBI-d and COMBI-v trials to report extended survival results showed overall survival of 34% at 5 years.^37^

The coBRIM trial compared the combination of vemurafenib and cobimetinib fumarate (*BRAF* and MEK inhibitors) with vemurafenib monotherapy for the treatment of *BRAF* V600–positive advanced melanoma and led to the approval of this combination in 2015 as well.^38^ The median PFS was better with the combination treatment (12.3 vs 7.2 months). The 5-year overall survival was also better with the combination treatment (31% vs 26%).^39^

In 2015, the OPTiM trial led to the approval of intralesional talimogene laherparepvec in patients with surgically unresectable stage III or IV melanoma.^40,41,42^ The durable response rate (lasting >6 months) was 16.3% in the talimogene laherparepvec group compared with 2.1% in the group receiving granulocyte-macrophage colony-stimulating factor. Median overall survival increased with longer follow-up to 23.3 months with talimogene laherparepvec and 18.9 months with granulocyte-macrophage colony-stimulating factor (HR, 0.79; *P* = .051).^40,41,42^

In 2018, the third combination of *BRAF* and MEK inhibitors—encorafenib and binimetinib—were approved for unresectable or metastatic melanoma based on the result of the COLUMBUS trial.^43^ The results of this phase 3 study found the median overall survival of the group receiving combination therapy to be 33.6 months compared with 16.7 months for vemurafenib (HR, 0.54 [95% CI, 0.40-0.71]).

In 2019, the CheckMate 511 study was conducted to address the toxicity of checkpoint inhibitor combination.^55^ This study compared FDA-approved nivolumab, 1 mg/kg, plus ipilimumab, 3 mg/kg, with nivolumab, 3 mg/kg, plus ipilimumab, 1 mg/kg. The latter combination demonstrated an improved safety profile, and there were no meaningful efficacy differences between the groups, even in the updated 3-year follow-up data. Although this dose is not yet FDA approved, it has been clinically adapted in various institutes as a preferred combination dose due to its better toxicity profile.

In 2020, atezolizumab (immunotherapy consisting of a programmed cell death ligand 1 [PD-L1] inhibitor) in combination with cobimetinib and vemurafenib was given to patients with *BRAF* V600 mutation–positive melanoma.^44^ Based on the results of the IMspire150 study, the FDA approved the combination of atezolizumab, vemurafenib, and cobimetinib therapy for metastatic melanoma. The phase 3 study found the triplet combination therapy to have a better median PFS of 15.1 vs 10.6 months (HR, 0.78; *P* = .02). The median overall survival was not mature.

Most recently, in March 2022, the FDA approved nivolumab and relatlimab, a LAG-3–blocking antibody for patients with unresectable or metastatic melanoma.^18^ The approval was based on results of the randomized phase 3 RELATIVITY-047 clinical trial (combination group vs nivolumab) in patients with previously untreated advanced melanoma. The median PFS was 10.1 months with relatlimab and nivolumab compared with 4.6 months with nivolumab (HR, 0.75; *P* = .006). The overall survival data are not mature yet. Grade 3 or 4 DRAE occurred in 18.9% of patients in the relatlimab-nivolumab group and in 9.7% of patients in the nivolumab group.

Unlike metastatic therapy approvals that began around the 1970s, adjuvant therapies for melanoma were not approved until 2011. Pegylated interferon alfa-2b was the first drug to be approved in the adjuvant setting for American Joint Committee on Cancer stage III melanoma in 2011 based on recurrence-free survival (RFS) advantage of 45.6% vs 38.9% at 4 years.^45^ No overall survival advantage was seen. With more effective immunotherapy approvals since, this therapy is not typically used.

In 2015, ipilimumab monotherapy (10 mg/kg for 4 doses) was approved in the adjuvant setting for patients with pathologic involvement of regional lymph nodes (>1 mm) after surgery.^46^ This approval was based on the EORTC (European Organisation for Research and Treatment of Cancer) 18071 trial that showed a longer median RFS with ipilimumab (26.1 vs 17.1 months; *P* = .001). At a high dose, adjuvant ipilimumab therapy was associated with significant toxicity; a 53% discontinuation rate was reported, and 5 patients (1%) died owing to DRAE. This was not readily used due to toxicity.^46^ Longer-term follow-up showed a better 7-year overall survival rate of 60.0% with ipilimumab vs 51.3% with placebo.^47^ Reducing the dose from 10 mg/kg to 3 mg/kg of body weight lowered the discontinuation rate while maintaining similar efficacy.^48^

In 2017, nivolumab (3 mg/kg) was approved based on favorable results seen in CheckMate 238 study compared with ipilimumab (10 mg/kg). The 12-month RFS was 70.5% with nivolumab and 60.8% with ipilimumab (HR, 0.65; *P* < .001).^49^ In a 4-year update from the study, no overall survival advantage was seen. However, a continued RFS benefit of 52% vs 41% (HR, 0.71; *P* < .001) was seen.^50^

Soon after, in 2017, the combination of dabrafenib plus trametinib was approved based on the results of the COMBI-AD trial as adjuvant therapy for patients with *BRAF* V600 mutation–positive stage III melanoma.^51^ The 3-year RFS was 58% with the combination therapy and 39% with placebo. The combination also improved overall survival at 3 years (86% vs 77%; HR, 0.57 [95% CI, 0.42-0.79]).^51^

Pembrolizumab was first approved by the FDA for use as adjuvant therapy for melanoma in 2019 for patients with stage III melanoma after complete resection based on the results of the Keynote-054 study.^52^ The 1-year RFS rate was 75.4% with pembrolizumab vs 61.0% with placebo (HR 0.57; *P* < .001). The benefit was seen regardless of PD-L1 status.^52^ Follow-up at 3.5 years continued to show RFS in favor of pembrolizumab at 59.8% vs 41.1% (HR, 0.59 [95% CI, 0.49-0.70]).^53^

More recently in 2021, pembrolizumab was approved as adjuvant monotherapy for completely resected high-risk stage II disease (stage IIB or IIC melanoma) after results of the phase 3 KEYNOTE-716 study.^54^ In a second interim analysis of this study at 21 months, RFS was not reached in either group, but fewer patients treated with pembrolizumab had a first recurrence of disease or mortality (15%) when compared with placebo (24%) (HR, 0.61 [95% CI, 0.45-0.82]).^54^ In patients with melanoma and *BRAF* V600 mutation, the most effective sequence of melanoma treatment using targeted therapy and immunotherapy continues to be investigated.^56,57,58^

## Discussion

In this study, we reviewed MMR trends in the US population during the last 4 decades, specifically 1975 to 2019, focusing on MMR with the changing melanoma treatment paradigm. After the introduction of newer treatments in 2011 (mostly after 2013), a significant reduction in MMR was seen from 2013 to 2017 in the US. This likely reflects a benefit from the availability of effective therapies in the last decade. The fact that even the elderly population (eg, those aged >75 years) was living longer with melanoma supports these therapies being well tolerated and successfully used in an elderly population.

Despite all these important advances in the treatment of melanoma, barriers to optimal care and accessibility to treatments remain an issue for many patients (eg, in rural areas).^59^ Also, the cost of modern therapies can be prohibitive for universal treatment access. Future directions need to address treatment access issues in addition to the development of new agents.

### Strengths and Limitations

The strength of our study is its large sample size from the publicly available SEER database, which is generalizable to the US population. These data include older and socioeconomically disadvantaged patients who are not very well represented in most randomized clinical trials.

This study, however, has limitations. Being a retrospective study, it is prone to unaddressed confounders as well as inherent selection bias from the SEER database and lacks a control population. The SEER database does not have information on clinical and chemotherapeutic data for individual patients that, if available, could be used to draw more definitive conclusions.

## Conclusions

The effectiveness of new and innovative therapeutics for melanoma is likely represented by the remarkable and statistically significant drop in population-level melanoma mortality in the last decade. These are very encouraging data and support the continued development of such therapies. Ways to address the accessibility of these treatments and the health care costs need to be supported.

## Notes

Supplement.
Data Sharing Statement
