# Supplementary material for: Melanoma Treatments and Mortality Rate Trends in the US, 1975 to 2019
Source: JAMA Netw Open. 2022 Dec 6;5(12):e2245269. doi: 10.1001/jamanetworkopen.2022.45269 (PMC9856246; doi:10.1001/jamanetworkopen.2022.45269)
Supplement: Supplement. — Data Sharing Statement [file jamanetwopen-e2245269-s001.pdf]

Data Sharing Statement

Kahlon. Melanoma Treatments and Mortality Rate Trends in the US, 1975 to 2019. *JAMA Netw Open*. Published December 06, 2022. doi:10.1001/jamanetworkopen.2022.45269

Data
Data available: No
